# Supplementary material for: S-1 and 5-Fluorouracil-related adverse events in patients with advanced gastric cancer: A meta-analysis
Source: PLoS One. 2023 Aug 11;18(8):e0290003. doi: 10.1371/journal.pone.0290003 (PMC10420378; doi:10.1371/journal.pone.0290003)
Supplement: Attachment — Submitted filename: Response to Reviewers.docx [file pone.0290003.s002.docx]

Additional Editor Comments:

The manuscript evaluated the adverse effects of two chemotherapy agents, suggesting that S-1 should be preferred in the treatment of gastric cancer.

There are some details in the manuscript that need to revise, such as the literature in the risk assessment map should be arranged by year. In addition, the formatting of the manuscript is very terrible, especially for the layout of figures and tables, which need to be completely revised.

Response: We revised the literature, figures and tables according to your suggestion.

Reviewer #1: Although the study was of great significance to explain the adverse events or reactions of 5-FU and S-1, the methods and reports of the manuscript did not meet the purpose of the study. My comments are as follows.

1. The review only included RCT and other types of studies such as non-randomized trials and case series reports were not collected. According to the PRISMA harms statement, these types of research advantages are important data sources.

Response: Due to the lack of scientific and reasonable control group, and easy to lead to misleading or deviation, it is not appropriate to conduct meta-analysis of non-randomized trials and case series.

2. Only adverse events were reported in the review. Adverse events need to analyze their causality with 5-FU and S-1. More importantly, in addition to the adverse events, these adverse reactions should also be reported and analyzed.

Response: Adverse events in our manuscript have included adverse reactions. Thanks for you comment.

Reviewer #2: The manuscript evaluated the adverse effects of two chemotherapy agents, suggesting that S-1 should be preferred in the treatment of gastric cancer. It has certain significance to guide the clinical use of chemotherapy agents. But, some issues should be mentioned.

1. The retrieval dates of the include literature in the manuscript need to be updated to ensure the rigor of the manuscript and the accuracy of the data included.

Response: According to your suggestion, we retrieval the literature up to March 31st, 2023. However, we failed to find new literature that meet the inclusion criteria of our manuscript.

2. Has the PROSPERO registration been conducted for this Meta-analysis? If so, please provide the registration number.

Response: This meta-analysis is registered in the PROSPERO, and the registration number is CRD42023409814. Thanks for your suggestion.

3. By reading the entire manuscript, it is only a summary of the included literature data and does not reflect the novelty of the research method or content. It is suggested that the author should be reconsider and revise the manuscript.

Response: Nursing can play an important role in controlling and alleviating adverse events caused by chemotherapy. This manuscript provides valuable reference for effectively controlling adverse events caused by chemotherapy and implementing effective nursing measures, which is an innovation of this manuscript.

4. It is suggested that the author should be optimized the title of each part to make the logic of the results and the description of primary and secondary indicators clearer.

Response: It is revised. Thanks for you advised.

5. There are some details in the manuscript that need to revise, such as the literature in the risk assessment map should be arranged by year. In addition, the formatting of the manuscript is very terrible, especially for the layout of figures and tables, which need to be completely revised.

Response: Thanks for your advise. The literature, figures and tables have revised.
