# Supplementary material for: S-1 and 5-Fluorouracil-related adverse events in patients with advanced gastric cancer: A meta-analysis
Source: PLoS One. 2023 Aug 11;18(8):e0290003. doi: 10.1371/journal.pone.0290003 (PMC10420378; doi:10.1371/journal.pone.0290003)
Supplement: Attachment — Submitted filename: Response to Reviewers.docx [file pone.0290003.s003.docx]

Review Comments to the Author

Reviewer #1:

Although the author has made revisions, the manuscript still has obvious shortcomings.

1. The authors have reported the results and methods used for assessing the risk of bias in individual studies. However, the risk of bias assessment should be considered separately for outcomes of benefit and harm.

Response:

In this work, the methods used to assessing the risk of bias in individual studies have been stated on page seven, lines 103 to 106, and the results of deviation assessment for individual studies have shown at Figs 2, 3 and 4. The purpose of bias evaluation is to test the quality of the included studies, which is used to confirm whether the reliability of the conclusion of meta-analysis is trustworthy. In addition, it is well known that the quality of included studies is positively related to the reliability of conclusions. The quality of the included studies in this meta-analysis is relatively high, so the conclusion of this work is highly reliable.

2. For adverse events analysis, inclusion in randomized controlled trials is one-sided and insufficient.

Response:

Due to the lack of scientific and reasonable control group, other types of studies (such non-randomized trials, case reports and so forth) are likely lead to misdirection or bias and not suitable for conducting meta-analysis.

3. The characteristics of the included studies remain underreported (Table 2). Some characteristics including dose, frequency, and course of treatment were not mentioned in the table.

Response:

Dose, frequency and course of treatment have been added into Table 2. Thanks for your advice.

4. Conjunctive therapy use of other drugs (Cisplatin, Paclitaxel) was also seen in the two groups in the included studies. The evaluation of the association of adverse events with interventions may be more complex. Also, it does not describe any assessment of possible causality.

Response:

Conjunctive therapy use of other drugs (Cisplatin, Paclitaxel) was also seen in the two groups in the included studies, which means there is comparability between S-1 and 5-FU groups. In addition, the aim of this study is to reveal the occurrence of adverse events caused by S-1 and 5-FU respectively. Therefore, the assessment of possible causality is another issue to be studied in the future.

Reviewer #2:

The manuscript evaluated the adverse effects of two chemotherapy agents, suggesting that S-1 should be preferred in the treatment of gastric cancer. It has certain significance to guide the clinical use of chemotherapy agents. This work has been adequately revised. It could be accepted for publication.

Response:

Thank you very much for your comments and support.
